# Effect of family background on the educational gradient in lifetime fertility of Finnish women born 1940–50

**DOI:** 10.1080/00324728.2014.913807

**Published:** 2014-06-20

**Authors:** Jessica Nisén, Mikko Myrskylä, Karri Silventoinen, Pekka Martikainen

**Affiliations:** ^a^ University of Helsinki; ^b^ London School of Economics and Political Science

**Keywords:** birth rate, childlessness, cohort analysis, education, intergenerational transmission, within-family design, fixed-effects model

## Abstract

An inverse association between education and fertility in women has been found in many societies but the causes of this association remain inadequately understood. We investigated whether observed and unobserved family-background characteristics explained educational differences in lifetime fertility among 35,212 Finnish women born in 1940–50. Poisson and logistic regression models, adjusted for measured socio-demographic family-background characteristics and for unobserved family characteristics shared by siblings, were used to analyse the relationship between education and the number of children, having any children, and fertility beyond the first child. The woman's education and the socio-economic position of the family were negatively associated with fertility. Observed family characteristics moderately (3–28 per cent) explained the association between education and fertility, and results from models including unobserved characteristics supported this interpretation. The remaining association may represent a causal relationship between education and fertility or joint preferences that form independently of our measures of background.

## Introduction

Education is a major determinant of women's fertility at the individual level and may also contribute to changes in cohort fertility (Rindfuss et al. 1996; Andersson et al. 2009; Bhrolcháin and Beaujouan 2012). A key question related to differences among women in this respect is to what extent the education–fertility relationship is causal (Gustafsson and Kalwij 2006). Selection may also contribute to the association because there may be other factors influencing both education and fertility that explain it (Upchurch et al. 2002; Martín-García and Baizán 2006; Skirbekk et al. 2006; Tavares 2010). Several methods are suited to the study of this issue (see Moffitt 2005; Gustafsson and Kalwij 2006; Moffitt 2009). One could include a set of potential confounding variables in the regression models (Tavares 2010) or use simultaneous modelling techniques (Upchurch et al. 2002). Among the methods used to tackle the problem of potential confounders have also been quasi-experimental designs based on policy reforms such as an increasing amount of compulsory schooling (Monstad et al. 2008), the marginal effect of the school leaving age (Skirbekk et al. 2006), and sibling and twin models (Geronimus and Korenman 1992; Kohler and Rodgers 2003; Kohler et al. 2010).

One possible source of confounding is family background (see Thornton 1980; Miller 1992, 1994; Axinn et al. 1996; Parr 2005; Rijken and Liefbroer 2009), but its nature and its importance in explaining the educational gradient in fertility is not well understood. We analysed the possible effect of family background on educational differences in lifetime fertility using a large, representative register-based cohort of Finnish women born in the period 1940–50, with lifetime fertility follow-up, non-retrospective information on early life environment, and links to identify family members (Statistics Finland 1997a). In order to do this we assessed the contribution of a rich set of measured socio-demographic family-background characteristics, reported in 1950, on the association between education and fertility. We were also able to control for the unobserved family-background factors shared by female siblings identified in the data. Both these methods have strengths and weaknesses, and by applying both we hoped to make the most of the former and reduce the effect of the latter.

The total lifetime number of children was treated as the main outcome variable in the analysis. Given that the life-course processes that lead to having the first child as opposed to subsequent children are likely to be different (see Kravdal 2001, 2007; Kreyenfeld 2002; Rønsen 2004; Vikat 2004), we also conducted separate analyses of the fertility outcomes of having any children and the number of children beyond the first child.

We used standard Poisson and logistic regression models and family fixed-effects specifications of these models to find out whether the associations between sisters were similar to those found between women in the whole cohort. The fixed-effects model controls for characteristics shared by sisters, which refer primarily to the family's social environment, but also to some genetically inherited characteristics. This method has been used previously in analyses of young-age parenthood and educational outcomes (e.g., Geronimus and Korenman 1992; Hoffman et al. 1993; Ribar 1999; Hofferth et al. 2001; Holmlund 2005). The model adjusts for selective characteristics common to sisters within a family, but the characteristics they do not share and which affect both fertility and education will still confound the estimates (Holmlund 2005; Kohler et al. 2010; Lahey and D'Onofrio 2010).

### Theoretical framework

A higher educational level among women is typically related to lower lifetime fertility (e.g., Rindfuss et al. 1996; Toulemon and Lapierre-Adamcyk 2000; Hoem et al. 2006a; Weeden et al. 2006; Fieder and Huber 2007; Kneale and Joshi 2008; Nettle and Pollet 2008), although the differences may be smaller among more recent cohorts (Andersson et al. 2009; Rønsen and Skrede 2010).

Educational differences in lifetime fertility may be attributable to causal mechanisms in which the direction may be from education to fertility or vice versa, or to selective mechanisms (Billari and Philipov 2004; Hoem et al. 2006a, 2006b; Martín-García and Baizán 2006). A negative causal influence of education on fertility may run through several pathways. Fertility is low during educational enrolment, a fact which could be explained by the difficulty of finding time for both studying and parenting, a lack of financial resources, or social norms discouraging parenting before finishing education (Hoem 1986; Blossfeld and Huinink 1991; Lappegård and Rønsen 2005; Bhrolcháin and Beaujouan 2012). By postponing family formation, longer educational enrolment periods may result in lower lifetime fertility because women face biological constraints to having children at higher ages and because there may also be social norms that discourage childbearing at higher ages independently of fecundity problems (Rindfuss and Bumpass 1976; Hagestad and Call 2007; Keizer et al. 2008; Billari et al. 2011).

There may also be influences of education on fertility net of those explained by the period of enrolment. Education may influence life values and orientation, which can further affect fertility decisions. For example, the more highly educated may be less constrained by traditional norms and hold more individualistic values, which may further encourage them to seek fulfilment in life without children (Lestaeghe 1983; van de Kaa 1996). Finally, education may affect fertility negatively because of the higher opportunity costs of having children for more highly educated women (Becker 1991; Liefbroer and Corijn 1999; Gustafsson 2001; Bhrolcháin and Beaujouan 2012), or alternatively because highly educated parents have higher expectations for their children that may increase the perceived costs of raising a child (Becker and Lewis 1973; Becker 1991).

On the other hand, some effects of education may have a positive impact on fertility: by leading to increased income, higher education may increase fertility by facilitating the formation of a family and childbearing (Becker 1991; Oppenheimer 1997; Liefbroer and Corijn 1999; Gustafsson 2001; Vikat 2004; Jalovaara and Miettinen 2013). Education may also increase fertility if it increases the stability of partnerships, in that stable partnerships are associated with higher fertility levels (Lyngstad and Jalovaara 2010; Jalovaara 2012).

Alternatively, the causal relationship between educational level and fertility may be in the reverse direction, from fertility behaviour to educational attainment (Hoffman et al. 1993; McElroy 1996; Hofferth et al. 2001; Cohen et al. 2011). Higher educational goals may be compromised as a consequence of having children because both parenting and studying are time-consuming and thus potentially competing activities (Dearden et al. 1995; Woodward et al. 2006). Furthermore, the early timing of fertility may affect further fertility by reducing the accumulation of human capital: if childbirth at a young age inhibits the mother from pursuing further educational or occupational achievements, continuing concentration on family life may be more rewarding relative to other opportunities in life (Morgan and Rindfuss 1999). Because a large share of teenage births can be expected to be unplanned (Henshaw 1998; Vikat et al. 2002), it can be argued that unplanned rather than planned births at young ages assert negative influences on further education.

In addition to the causal mechanisms that may run both ways, selective mechanisms may also contribute to educational differences in fertility. Some rather stable preferences could influence women's choices in ways that affect both family and working life (Hakim 2000). Possibly the strongest empirical evidence for selective mechanisms comes from studies on very young mothers, who tend to end up with low educational attainment: some studies attribute this largely to selection and not to the influence of childbearing on education, but the evidence in general is inconsistent (e.g., Geronimus and Korenman 1992; Hoffman et al. 1993; Ribar 1999; Hofferth et al. 2001; Lee 2010). Furthermore, studies of the progression to higher-order parities reveal that unobserved factors that influence previous transition(s), such as family preferences or fecundity, may play a role in the educational differences in higher-order transitions, even if these factors did not correlate with education at the beginning of the reproductive process (Kravdal 2001; Kreyenfeld 2002).

Family background is a potentially relevant factor influencing the education–fertility association because factors clustered in the families of origin may influence preferences and constraints on family life and educational choices (see Thornton 1980; Miller 1992, 1994; Axinn et al. 1996). Interaction between genetic predispositions and characteristics of the social environment in childhood and adolescence may affect the motivation to have children (Miller 1992, 1994). Life goals other than family building, such as having a career, might be emphasized more strongly in families in which parents have a higher socio-economic status (Scott 2004; Rijken and Liefbroer 2009), and the potential influence may extend to attitudes and behavioural outcomes in the next generation.

The material resources of the family of origin may also influence the consumption aspirations of the next generation, the members of which may strive for a higher economic standing through education before or instead of having children (Easterlin 1966; Thornton 1980). An advantaged family background may also allow prolonged schooling through the provision of resources, whereas a less advantaged background may not. Other factors in the family background that may influence fertility and education include sibship size (Murphy and Knudsen 2002; Pouta et al. 2005), the mother's fertility preferences (Axinn et al. 1996), and urban rather than rural residence (Lestaeghe 1983; Kulu et al. 2007). Overall, it seems plausible that family-background factors exert an influence on both education and fertility, and thus contribute to the association between the two.

Finland was a poor country, at or recovering from war in 1940–50, when the women we studied were born and spent the early years of their life (Jäntti et al. 2006). Later, in the second half of the century, the country went through profound changes, including a rising level of overall living standards. It has long had a relatively gender-neutral labour market: the labour force participation of women with children has been high and the share of women working part-time very low (Rønsen and Sundström 2002). A universal right to paid family leave in Finland dates back to 1964. During subsequent decades the length of the leave was extended several times (from 9 weeks in 1964 to 44 weeks in 1987) and the income replacement level was also raised substantially (Rønsen 2004). At the turn of the century the country could be characterized as a Nordic welfare state with generous family benefits and a high level of gender equality (Rønsen and Skrede 2010).

## Data and methods

### Data

The data were obtained from a 10-per-cent sample of households drawn from the 1950 Finnish Census of Population (Statistics Finland 1997a). Information on individuals who belonged to the sampled households was subsequently linked to socio-demographic information from quinquennial censuses from 1970 to 1995, and to the Finnish Population Register for fertility histories. We restricted the data to the 1940–50 birth cohorts. The original sample consisted of 411,628 persons of whom 91,452 were born between 1940 and 1950 and lived in a one-parent or two-parent family at the time of the census in 1950. The sample included 44,672 women. We excluded from the study sample respondents with missing information on family-background variables, those not present in the census at the age of 30–34 (*n* = 8,413), and those lost to follow-up at the age of 45–49 (*n* = 1,047). Loss to follow-up is attributable to emigration, mainly to Sweden in the late 1960s and early 1970s, and to a lesser extent to mortality between 1950 and 1990–95. This left us with the final study sample of 35,212 women. Sisters were identified on the basis of an identification code collected in 1950 for place of residence, household, and family. The women in the sample came from 26,207 families altogether, in 6,979 of which at least two female siblings could be identified. This identification procedure does not make any distinction between biological and non-biological sisters.

Information on live births was linked to the data via the personal identification numbers given to all Finnish citizens by the late 1960s. Children born before 1970 were registered to their mothers conditional on co-residence at the time of the 1970 population census. The original fertility information consisted of links to both biological and adopted children, but here we took into account only the biological links in order to measure the number of biological children born to the study participants. This procedure eliminated very few mother–child links: excluding non-biological children decreased the overall lifetime fertility by only 0.01 children and the proportion of mothers by only 0.5 percentage points. We used three fertility measures. The main outcome variable was the lifetime number of children. The study participants were aged 59 or older at the end of the follow-up in 2009, which means that their fertility was truly complete and no truncation occurred. The analysis also covered having any children (vs. childlessness) and the number of children beyond the first child.

The main explanatory variable, the level of education, was measured at the age of 30–34 and categorized into four classes: basic, lower secondary, upper secondary, and tertiary (Table 1). The basic level refers to a maximum of 9 years of general education (9 years or less). The lower-secondary level refers to brief vocational training (<3 years) undertaken in addition to general education. Upper-secondary education refers to either academic education (matriculation) or vocational training (≥3 years) undertaken in addition to general education. Finally, the tertiary level refers either to a university degree or to vocational training at the highest level (such as for specialized nurses and elementary school teachers) (≥4 years after general education). Some vocational qualifications that were counted as tertiary-level degrees in later classifications (Statistics Finland 1997b) are included in the secondary level in this classification from 1988 (Statistics Finland 1989).

**Table 1:** Descriptive statistics. Finnish women born in 1940–50, *N* = 35,212

	*N* of children		Having any children		*N* of children beyond the first one^1^	
*Lifetime fertility by level of education*	M	SD	%	*N*	M	SD
Level of education						
Basic	1.94	1.36	86.1	13,958	1.26	1.21
Lower secondary	1.83	1.30	84.8	7,991	1.16	1.11
Upper secondary	1.73	1.26	81.6	4,269	1.12	1.14
Tertiary	1.73	1.43	78.5	3,404	1.20	1.19
Total	1.85	1.38	84.1	29,622	1.20	1.20
*Explanatory variables: level of education and family-background characteristics*						
	*N*	%			*N*	%
Level of education			House ownership			
Basic	16,216	46.1	Owner		21,041	59.8
Lower secondary	9,429	26.8	Renter		12,106	34.4
Upper secondary	5,231	14.9	Other, unknown		2,065	5.9
Tertiary	4,336	12.3				
			Crowding (*n* of persons/heated room)			
Parental level of education			<2		11,379	32.3
Less than primary	5,113	14.5	2<3		11,569	32.9
Primary	26,433	75.1	3<4		5,758	16.4
More than primary	3,666	10.4	≥4		6,506	18.5
Occupational status of the family head			Standard of living			
Professional/administrative	5,488	15.6	Poor		10,099	28.7
Manual worker	14,937	42.4	Modest		16,243	46.1
Farmer, <10 hectares	8,910	25.3	Good		8,870	25.2
Farmer, ≥10 hectares	2,728	7.8	Living area			
Self-employed, other, unknown	3,149	8.9	Helsinki region		2,616	7.4
Number of siblings			Rest of Uusimaa		2,049	5.8
0	5,200	14.8	Western Finland		13,942	39.6
1–2	16,946	48.1	Eastern Finland		15,094	42.9
3–	13,066	37.1	Northern Finland		1,511	4.3
Family type						
Two parents and children	32,693	92.9				
Mother and children	2,272	6.5				
Father and children	247	0.7				

^1^ Among mothers only.

*Source*: Data from the 1950 Finnish Census of Population linked to data from quinquennial censuses between 1970 and 1995 and to data on live births from Statistics Finland.

The socio-economic position of the family of origin included measures of parents' education and occupational status. The parents' educational level refers to the highest educational qualification achieved by either parent, categorized as less than primary school, primary school, and more than primary school. The occupational status of the family head was categorized as follows: professional/administrative, manual worker, farmer with <10 hectares (100 acres) of land, farmer with ≥10 hectares of land, and self-employed or other. Family-structure variables were family type (two parents with children, mother and children, father and children) and number of siblings (0, 1–2, 3–) living in the household in 1950. Three variables measured overall living conditions: house ownership (owner, renter, other or unknown); crowding (number of persons per heated room: <2, 2 < 3, 3 < 4, ≥4); and standard of living (poor, modest, good). In this proximate measure of standard of living, the category ‘poor’ referred to households with no modern facilities such as electric light, ‘modest’ to households with one item, and ‘good’ to those with at least two items. The area-of-residence variable covered five locations: the Helsinki (capital) region, the rest of Uusimaa (the area surrounding the capital region in the south part of Finland), Western Finland, and Eastern and Northern Finland, both of which were mainly agricultural areas in 1950. All these observed family-background variables measured conditions in 1950 at the time of the census, when the women analysed here were between the ages of 0 and 10.

### Statistical methods

We first estimated three standard regression models, without the fixed-effects specification, for each of the three fertility outcomes. These three models document the fertility–education associations and show the extent to which observed family-background characteristics explain them. Model 1 documents the associations between each single explanatory variable and the fertility outcomes, adjusted only for year of birth. All the measured family-background variables and the year of birth are simultaneously adjusted for in Model 2, but the woman's own education is not included. Model 3 adds the level of education to Model 2. We used standard Poisson regression models to assess the relationship between education and the number of children. Correspondingly, binary logistic regression (having any children) and Poisson regression (the number of children beyond the first child) were used to study the association between fertility and the two other fertility outcomes. The sample used to estimate the models necessarily varied according to the outcome of these analyses: we used the full sample of women (*N* = 35,212) for the main fertility outcome and for having any children, but only included mothers when analysing the number of children beyond the first one (*n* = 29,622).

In the next stage, we used fixed-effects regression models to find out whether unobserved family-background characteristics contributed to the association between education and the fertility outcomes. This fixed-effects approach uses the family indicator to capture the unobserved family characteristics, and estimates the model parameters for adult characteristics—including education—from the variation between sisters. Thus, the models fully accounted for the shared family environment, but at the cost of reducing the sample size because those who had no sisters were excluded. Further, the sister sets in which all sisters remained childless were excluded in the Poisson fixed-effects models (number of children/number of children beyond the first one), and sets in which all sisters had the same outcome were excluded in the logistic fixed-effects regression model (of having any children). The analysed sister sets included sisters born between 1940 and 1950, and who were alive and living in the same household in 1950 at the time of the census.

Because of the restrictions to the analysed sample when moving from the standard models to the fixed-effects models, the two sets of results were not directly comparable. Therefore, when we conducted the fixed-effects analyses we first re-estimated Model 1 (education–fertility association adjusted only for the year of birth) and Model 3 (full adjustment for observed family characteristics and the year of birth) using only the subsample of women with one or more sisters present in the analysed sample, as explained above (otherwise irrespective of their fertility/education outcomes). We then estimated the fixed-effects model, Model 4, which controlled for the year of birth and, through the fixed effects, for all observed and unobserved family-background characteristics shared by the sisters. In practice, however, only the sets varying in both education and fertility within the set contributed to the fixed-effects estimates of the education coefficient.

We constructed the family fixed-effects models by means of conditional maximum likelihood estimation (Allison 2009). Throughout the analysis we accounted for the clustering of sisters within families in the calculation of the 95-per-cent confidence intervals (CIs) and other variance-based measures (but not in the calculation of the AICs). We used the bootstrap procedure with cluster resampling in calculating the CIs, with 1,000 replications and sibling sets as the clusters (Carpenter and Bithell 2000). The results of the Poisson regression models are reported as incidence-rate ratios (IRRs) and those of the binary logistic regression models as odds ratios (ORs). In addition, we present the results of the Poisson models in terms of the number of children, taking the age-adjusted fertility rate (number of children) in the reference group (women with a basic level of education) and multiplying it by the IRRs derived from the estimated model. The Stata statistical package, Version 11 (StataCorp 2009), was used for all the statistical analyses.

An alternative to the Poisson model specification would have been the negative binomial model, which would have been preferable had there been over-dispersion. We assessed this in all the standard models by means of likelihood ratio testing, and found no evidence of over-dispersion for the main fertility outcome. There was some evidence of over-dispersion with regard to the number of children beyond the first child (Model 1: alpha = 0.016, 95 per cent CI 0.004, 0.060). We therefore also ran these models with the negative binomial model, but the results showed no difference from the results of the Poisson model presented here. Furthermore, we ran all the models with ordinary least squares regression in addition to the preferred Poisson and logistic specifications, but the results changed only marginally.

## Results

### Descriptive results

Table 1 gives the descriptive statistics of the study population. The largest educational group, covering almost half of the sample (46 per cent), comprised women with a basic level of education. Of the rest, over a quarter (27 per cent) had reached the lower-secondary level, 15 per cent the upper-secondary level, and 12 per cent the tertiary level. Childlessness was already relatively common in this birth cohort: 16 per cent of the women had no children. The mean number of children born was 1.85 (SD 1.38), and among the women with children the mean number beyond the first one was 1.20 (SD 1.20).

A negative educational gradient is documented for number of children: the eventual average number for women with a basic education is 1.94, among those with lower-secondary education it is 1.83, and the number falls to 1.73 among those educated to the upper-secondary and tertiary levels. Moreover, having any children is less common among the more highly educated: 86 per cent of those with a basic education have children compared with only 79 per cent of those educated to the tertiary level. With regard to the number of children beyond the first one, a U-shaped association with educational level emerges: the women with an upper-secondary education have the fewest children (1.12).

The majority of women came from manual-worker (42 per cent) or farmer (33 per cent) families, and had parents with, at most, primary-school education (only 10 per cent had a mother or a father educated to a higher level). Few women came from one-parent families (7 per cent) and the vast majority (85 per cent) had at least one sibling. The families from which these women came typically lived in owner-occupied accommodation (60 per cent), with fewer than three persons per heated room (65 per cent) and a modest standard of living (46 per cent). The overwhelming majority came from Western or Eastern (83 per cent) Finland, which in 1950 was largely agricultural.

### Analytical results: the effect of observed family background

**Figure 1:**
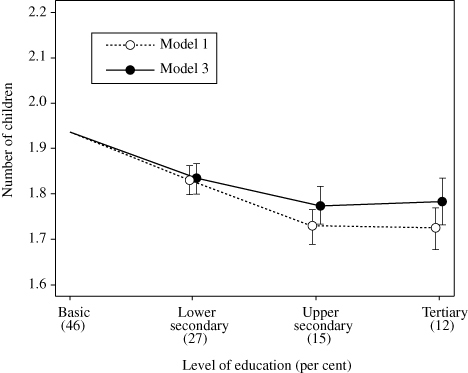
Number of children with 95 per cent CI by educational level among Finnish women born in 1940–50, *N* = 35,212. Results based on standard Poisson regression model: *Model 1*: year of birth + level of education
*Model 3*: year of birth + level of education + family-background characteristics.
*Source*: As for Table 1

As expected, there is a negative association between the women's educational level and the number of children (Table 2, Model 1): the IRR of those educated to the tertiary level is 0.89 (95 per cent CI 0.87, 0.91) compared to those on the basic level. In other words, those with tertiary education have approximately 11 per cent fewer children than those with basic education. Figure 1 illustrates this difference by number of children. The above results are proportioned to the birth-year-adjusted number of children in the latter group observed in the data (1.93). The 11-per-cent lower fertility of the former group then corresponds to 1.72 children (95 per cent CI 1.68, 1.77). The corresponding figures for the other educational groups are the following: upper secondary 1.73 (95 per cent CI 1.69, 1.77) and lower secondary 1.83 (95 per cent CI 1.80, 1.86).

**Table 2:** Number of children by level of education and family-background characteristics among Finnish women born in 1940–50. Poisson regression, IRR and 95 per cent CI,^1^ *N* = 35,212

	1		2		3	
Model	IRR	95% CI	IRR	95% CI	IRR	95% CI
*Level of education (Basic)*^2^						
Lower secondary	0.95	(0.93–0.96)			0.95	(0.93–0.96)
Upper secondary	0.89	(0.87–0.91)			0.92	(0.90–0.94)
Tertiary	0.89	(0.87–0.91)			0.92	(0.90–0.95)
*Parental level of education (Less than primary school)*						
Primary school	0.94	(0.92–0.97)	0.98	(0.96–1.00)	0.98	(0.96–1.01)
More than primary school	0.88	(0.85–0.91)	0.98	(0.94–1.02)	1.00	(0.96–1.04)
*Occupational status of the family head (Professional/administrative)*						
Workers	1.07	(1.04–1.10)	1.02	(0.99–1.05)	1.01	(0.98–1.04)
Farmers with <10 hectares	1.12	(1.09–1.15)	1.03	(0.99–1.06)	1.02	(0.98–1.05)
Farmers with ≥10 hectares	1.10	(1.06–1.14)	1.04	(1.01–1.09)	1.04	(1.00–1.08)
Self-employed, other, unknown	1.07	(1.03–1.11)	1.03	(0.99–1.06)	1.02	(0.99–1.06)
*Family type (Two parents with children)*						
Mother and children	0.98	(0.95–1.01)	1.00	(0.97–1.03)	1.00	(0.97–1.03)
Father and children	0.98	(0.88–1.08)	0.97	(0.87–1.08)	0.96	(0.87–1.07)
*Number of siblings (0)*						
1–2	1.03	(1.01–1.05)	1.02	(1.00–1.04)	1.02	(1.00–1.04)
3–	1.11	(1.08–1.14)	1.06	(1.03–1.09)	1.05	(1.03–1.08)
*House ownership (Owner)*						
Renter	0.93	(0.91–0.94)	0.97	(0.95–0.99)	0.97	(0.95–0.99)
Other, unknown	0.97	(0.94–1.01)	0.98	(0.94–1.01)	0.98	(0.94–1.01)
*Crowding (n of persons/heated room) (<2)*						
2<3	1.03	(1.01–1.04)	1.00	(0.98–1.03)	1.00	(0.98–1.02)
3<4	1.06	(1.04–1.09)	1.02	(1.00–1.05)	1.01	(0.99–1.04)
≥4	1.10	(1.07–1.12)	1.05	(1.02–1.08)	1.04	(1.01–1.07)
*Standard of living (Poor)*						
Modest	0.95	(0.93–0.97)	0.99	(0.96–1.01)	0.99	(0.97–1.01)
Good	0.89	(0.87–0.91)	0.96	(0.94–0.99)	0.97	(0.95–1.00)
*Living area (Helsinki region)*						
Rest of Uusimaa	1.06	(1.02–1.10)	1.02	(0.98–1.06)	1.02	(0.98–1.06)
Western Finland	1.10	(1.07–1.13)	1.05	(1.02–1.08)	1.05	(1.02–1.09)
Eastern Finland	1.14	(1.10–1.17)	1.07	(1.03–1.10)	1.07	(1.04–1.11)
Northern Finland	1.21	(1.15–1.26)	1.12	(1.07–1.17)	1.14	(1.08–1.19)
*Wald χ*^2^*, df, p*	160.8, 4, <0.001^3^		271.1, 22, <0.001		383.8, 25, <0.001	
*AIC*	114,095.4^3^		114,018.9		113,952.5	

*Model 1*: year of birth + variable.

*Model 2*: year of birth + family-background characteristics.

*Model 3*: year of birth + level of education + family-background characteristics. (This model adds family-background characteristics to Model 1 for the women's own level of education.)

^1^ The bootstrap procedure was used to calculate the confidence intervals.

^2^ Omitted category in parentheses.

^3^ The statistical test refers to the model for the women's own level of education.

*Source*: As for Table 1.

Several of the socio-demographic family-background variables also turn out to be associated with number of children. For example, a higher level of parents' education predicts lower fertility: the IRR of women whose parents have more than a primary-school education is 0.88 (95 per cent CI 0.85, 0.91) compared to those whose parents are not educated up to the primary-school level. Women from manual-worker and small or large farmer families have more children than those from families in which the occupational status of the head is professional or administrative. Family type is not associated with the women's fertility, but those with more siblings have more children. Women living in rented housing in 1950 have fewer children than those living in owner-occupied dwellings, whereas those living in more crowded dwellings have more children, as do those with a lower standard of living in childhood. Living in the capital region (Helsinki) predicts lower fertility than living in less affluent and more heavily agricultural areas. Adding all the family-background characteristics into the model simultaneously (Model 2) clearly attenuates all the effects, in particular that of parents' education.

The woman's own level of education was incorporated into Model 3. Compared with Model 1, adjustment for the family-background variables moderately, by approximately a quarter, attenuates the educational gradient in fertility (tertiary education: IRR 0.92 95 per cent CI 0.90, 0.95). In terms of the number of children (Figure 1), following adjustment for the measured family-background characteristics the difference between women with tertiary and those with basic education decreases from 0.21 to 0.15 children. The difference between women with upper secondary and those with basic level of education is similarly attenuated, but remains unchanged among those with lower-secondary level. According to additional analyses, if parents' education and occupational status are controlled for first, further adjustments for other variables have little effect (the results are not shown but are available on request). The associations between the observed family-background variables and the number of children do not change much following adjustment for the women's own educational level.

We conducted corresponding analyses for the two other fertility outcomes—having any children (Table 3) and the number of children beyond the first child (Table 4). In general, the associations with the explanatory variables are in the same direction for having any children (Table 3, Model 1). The women's own level of education is negatively associated with having any children: the OR of those with a tertiary level of education is 0.59 (95 per cent CI 0.54, 0.64) compared to those with a basic level. Family background is also predictive of having any children: women from manual-worker and farmer families are more likely to have children than those from families with a head in an administrative or professional occupation. Following adjustment for the family-background variables it turns out that area of residence, a manual-worker background, and parents' education predict having any children, net of the other background variables (Model 2). The adjustment has hardly any effect on the association between the women's own education and having any children: the OR of those educated to the tertiary level is 0.63 (95 per cent CI 0.58, 0.69) compared to those with a basic education (Model 3). Adjustment for educational level weakly attenuates the differences in having any children by parents' education and occupational status, but not by area of residence.

**Table 3:** Having any children by level of education and family-background characteristics among Finnish women born in 1940–50. Logistic regression, OR and 95 per cent CI,^1^ *N* = 35,212

	1		2		3	
Model	OR	95% CI	OR	95% CI	OR	95% CI
*Level of education (Basic)*^2^						
Lower secondary	0.89	(0.83–0.96)			0.89	(0.83–0.96)
Upper secondary	0.71	(0.65–0.77)			0.74	(0.67–0.80)
Tertiary	0.59	(0.54–0.64)			0.63	(0.58–0.69)
*Parental level of education (Less than primary school)*						
Primary school	0.96	(0.87–1.04)	1.01	(0.91–1.10)	1.03	(0.94–1.13)
More than primary school	0.68	(0.61–0.76)	0.85	(0.74–0.97)	0.95	(0.82–1.08)
*Occupational status of the family head (Professional/administrative)*						
Workers	1.36	(1.25–1.47)	1.17	(1.07–1.30)	1.09	(0.99–1.21)
Farmers with <10 hectares	1.34	(1.22–1.48)	1.09	(0.98–1.23)	1.03	(0.92–1.16)
Farmers with ≥10 hectares	1.21	(1.07–1.38)	1.03	(0.91–1.22)	0.99	(0.87–1.17)
Self-employed, other, unknown	1.25	(1.11–1.41)	1.11	(0.97–1.27)	1.06	(0.93–1.21)
*Family type (Two parents with children)*						
Mother and children	1.00	(0.89–1.12)	1.03	(0.91–1.17)	1.01	(0.90–1.15)
Father and children	0.76	(0.56–1.09)	0.75	(0.55–1.08)	0.73	(0.54–1.04)
*Number of siblings (0)*						
1–2	1.09	(1.01–1.20)	1.09	(0.99–1.20)	1.08	(0.99–1.19)
3–	1.12	(1.08–1.30)	1.11	(1.00–1.21)	1.08	(0.97–1.18)
*House ownership (Owner)*						
Renter	0.89	(0.84–0.95)	0.97	(0.90–1.05)	0.97	(0.90–1.05)
Other, unknown	0.97	(0.85–1.10)	0.98	(0.86–1.12)	0.98	(0.86–1.12)
*Crowding (n of persons/heated room) (<2)*						
2<3	1.10	(1.03–1.19)	1.00	(0.93–1.09)	0.97	(0.90–1.06)
3<4	1.12	(1.02–1.23)	0.99	(0.90–1.10)	0.95	(0.86–1.06)
≥4	1.22	(1.12–1.34)	1.06	(0.95–1.17)	1.00	(0.90–1.12)
*Standard of living (Poor)*						
Modest	0.93	(0.87–1.00)	0.96	(0.88–1.04)	0.98	(0.90–1.06)
Good	0.77	(0.71–0.83)	0.89	(0.80–0.99)	0.95	(0.84–1.05)
*Living area (Helsinki region)*						
Rest of Uusimaa	1.26	(1.07–1.47)	1.13	(0.94–1.32)	1.12	(0.94–1.32)
Western Finland	1.33	(1.19–1.48)	1.18	(1.04–1.32)	1.20	(1.06–1.34)
Eastern Finland	1.32	(1.19–1.47)	1.12	(0.99–1.26)	1.15	(1.01–1.29)
Northern Finland	1.59	(1.34–1.91)	1.34	(1.11–1.62)	1.40	(1.16–1.69)
*Wald χ*^2^*, df, p*	179.8, 4, <0.001^3^		116.0, 22, <0.001		230.4, 25, <0.001	
*AIC*	30,657.2^3^		30,747.2		30,651.2	

*Model 1*: year of birth + variable.

*Model 2*: year of birth + family-background characteristics.

*Model 3*: year of birth + level of education + family-background characteristics. (This model adds family-background characteristics to Model 1 for the women's own level of education.)

^1^ The bootstrap procedure was used to calculate the confidence intervals.

^2^ Omitted category in parentheses.

^3^ The statistical test refers to the model for the women's own level of education.

*Source*: As for Table 1.

**Table 4:** Number of children beyond the first one by level of education and family-background characteristics among Finnish mothers born in 1940–50. Poisson regression, IRR and 95 per cent CI,^1^ *N* = 29,622

	1		2		3	
Model	IRR	95% CI	IRR	95% CI	IRR	95% CI
*Level of education (Basic)*^2^						
Lower secondary	0.93	(0.91–0.95)			0.93	(0.91–0.96)
Upper secondary	0.90	(0.87–0.93)			0.93	(0.90–0.97)
Tertiary	0.96	(0.93–1.00)			1.00	(0.96–1.04)
*Parental level of education (Less than primary school)*						
Primary school	0.91	(0.88–0.94)	0.96	(0.93–0.99)	0.96	(0.93–1.00)
More than primary school	0.90	(0.86–0.94)	1.02	(0.97–1.08)	1.02	(0.97–1.08)
*Occupational status of the family head (Professional/administrative)*						
Workers	1.03	(0.99–1.06)	0.99	(0.95–1.04)	0.99	(0.95–1.04)
Farmers with <10 hectares	1.12	(1.08–1.16)	1.02	(0.97–1.07)	1.02	(0.97–1.07)
Farmers with ≥10 hectares	1.12	(1.07–1.18)	1.07	(1.01–1.13)	1.07	(1.01–1.14)
Self-employed, other, unknown	1.05	(0.99–1.09)	1.01	(0.96–1.06)	1.01	(0.96–1.06)
*Family type (Two parents with children)*						
Mother and children	0.97	(0.93–1.02)	0.99	(0.95–1.03)	0.99	(0.95–1.03)
Father and children	1.05	(0.91–1.21)	1.04	(0.90–1.19)	1.03	(0.89–1.19)
*Number of siblings (0)*						
1–2	1.03	(0.99–1.06)	1.01	(0.98–1.04)	1.01	(0.98–1.04)
3–	1.15	(1.12–1.19)	1.08	(1.04–1.12)	1.07	(1.04–1.12)
*House ownership (Owner)*						
Renter	0.90	(0.88–0.92)	0.95	(0.92–0.98)	0.95	(0.92–0.98)
Other, unknown	0.96	(0.91–1.01)	0.97	(0.92–1.01)	0.97	(0.92–1.01)
*Crowding (n of persons/heated room) (<2)*						
2<3	1.02	(0.99–1.05)	1.01	(0.97–1.04)	1.01	(0.98–1.04)
3<4	1.08	(1.05–1.12)	1.04	(1.00–1.08)	1.04	(1.00–1.08)
≥4	1.11	(1.08–1.16)	1.07	(1.03–1.11)	1.06	(1.02–1.11)
*Standard of living (Poor)*						
Modest	0.92	(0.90–0.95)	0.99	(0.95–1.02)	0.99	(0.95–1.02)
Good	0.87	(0.85–0.90)	0.96	(0.93–1.01)	0.97	(0.93–1.01)
*Living area (Helsinki region)*						
Rest of Uusimaa	1.03	(0.97–1.09)	0.99	(0.93–1.05)	0.99	(0.93–1.05)
Western Finland	1.09	(1.04–1.14)	1.03	(0.99–1.08)	1.04	(0.99–1.09)
Eastern Finland	1.17	(1.11–1.22)	1.08	(1.03–1.13)	1.09	(1.03–1.14)
Northern Finland	1.24	(1.16–1.32)	1.13	(1.05–1.21)	1.14	(1.07–1.23)
*Wald χ*^2^*, df, p*	77.8, 4, <0.001^3^		255.7, 22, <0.001		304.7, 25, <0.001	
*AIC*	81,844.5^3^		81,670.0		81,636.5	

*Model 1*: year of birth + variable.

*Model 2*: year of birth + family-background characteristics.

*Model 3*: year of birth + level of education + family-background characteristics. (This model adds family-background characteristics to Model 1 for the women's own level of education.)

^1^ The bootstrap procedure was used to calculate the confidence intervals.

^2^ Omitted category in parentheses.

^3^ The statistical test refers to the model for the women's own level of education.

*Source*: As for Table 1.

The analyses reveal a U-shaped association between number of children beyond the first child and the women's own level of education: the IRR of those educated to the tertiary level is 0.96 (95 per cent CI 0.93, 1.00) compared to those with a basic education (Table 4, Model 1). The women with an upper-secondary education have fewest children beyond the first child: the IRR is 0.90 (95 per cent CI 0.87, 0.93) compared to those with a basic education. The associations between family background and both fertility beyond the first child and the total number of children are similar in direction and strength. As with the analyses of the main outcome variable, several family-background characteristics remain significant predictors of fertility beyond the first child, net of other measured characteristics (Model 2). When the women's own level of education is included in Model 3, the measured family-background variables account for its association with fertility beyond the first child moderately compared to Model 1: the IRR of those with a tertiary and an upper-secondary education changes to 1.00 (95 per cent CI 0.96, 1.04) and 0.93 (95 per cent CI 0.90, 0.97), respectively. The women's own educational level does not appear to account for the effects of these variables on fertility beyond the first child.

### Analytical results: the effect of unobserved family background

**Figure 2:**
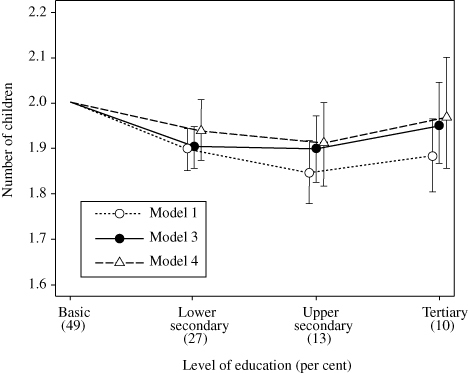
Number of children with 95 per cent CI by educational level in the subsample of Finnish women born in 1940–50, *n* = 15,746. Results based on standard and fixed-effects Poisson regression model: *Model 1*: year of birth + level of education
*Model 3*: year of birth + level of education + family-background characteristics
*Model 4*: year of birth + level of education + family fixed characteristics
*Source*: As for Table 1.

Next, we report the analyses of subsamples of women included in the family fixed-effects regression models (Table 5). The association between level of education and number of children is weaker in the subsample (*n* = 15,746) than among all the women: the IRR of those with a tertiary level of education is 0.94 (95 per cent CI 0.90, 0.98) (Model 1), whereas in the full sample and with the corresponding model it is 0.89 (95 per cent CI 0.87, 0.91). Figure 2 shows these results proportioned to the number of children—the fertility of the women with a basic education in this sample adjusted only for year of birth is 2.00 children. Assuming this baseline fertility, an IRR of 0.94 corresponds to 0.11 children fewer for the women educated to the tertiary level compared to those with only a basic education. Adjusting for the measured family-background characteristics in this sample (Model 3) further reduces the association somewhat: the IRR of those with a tertiary education is 0.98 (95 per cent CI 0.94, 1.02). The difference of 0.11 falls to 0.05 in number of children (Figure 2). Finally and according to the point estimates, small differences are observable in the fixed-effects model, which accounts for characteristics shared by sisters (Model 4), although they are no longer statistically significant: the IRR of those educated to the tertiary level is 0.98 (95 per cent CI 0.93, 1.05). This corresponds to a difference of only 0.03 in number of children.

**Table 5:** Lifetime fertility by level of education among Finnish women born in 1940–50. Standard and fixed-effects regression.

Model	1		3^4^		4	
*Number of children. Poisson regression, IRR and 95% CI*^1^*, N = 15,746*						
	IRR	95% CI	IRR	95% CI	IRR	95% CI
*Level of education (Basic)*^2^						
Lower secondary	0.95	(0.93–0.97)	0.95	(0.93–0.97)	0.97	(0.94–1.00)
Upper secondary	0.92	(0.89–0.96)	0.95	(0.91–0.99)	0.96	(0.91–1.00)
Tertiary	0.94	(0.90–0.98)	0.98	(0.94–1.02)	0.98	(0.93–1.05)
*Wald χ*^2^*, df, p*	37.7, 4, <0.001		152.9, 24, <0.001		28.6, 4, <0.001	
*AIC*	51,568.9		51,507.3		22,268.3	
*Having any children. Logistic regression, OR and 95% CI, N = 4,491*						
	OR	95% CI	OR	95% CI	OR	95% CI
*Level of education (Basic)*						
Lower secondary	0.90	(0.79–1.04)	0.91	(0.80–1.06)	0.91	(0.77–1.06)
Upper secondary	0.79	(0.67–0.93)	0.83	(0.69–0.99)	0.83	(0.68–1.04)
Tertiary	0.86	(0.74–1.00)	0.90	(0.74–1.07)	0.86	(0.69–1.11)
*Wald χ*^2^*, df, p*	12.8, 4, <0.01		161.1, 24, <0.01		3.8, 4, = 0.439	
*AIC*	6,194.7		6,221.2		3,193.5	
*Number of children beyond the first one. Poisson regression, IRR and 95% CI, N = 11,569*^3^						
	IRR	95% CI	IRR	95% CI	IRR	95% CI
*Level of education (Basic)*						
Lower secondary	0.94	(0.91–0.97)	0.94	(0.91–0.98)	0.97	(0.93–1.02)
Upper secondary	0.94	(0.89–1.01)	0.98	(0.92–1.05)	0.97	(0.90–1.05)
Tertiary	1.00	(0.93–1.07)	1.06	(0.98–1.15)	1.01	(0.92–1.11)
*Wald χ*^2^*, df, p*	26.7, 4, <0.001		113.4, 24, <0.001		34.3, 4, <0.001	
*AIC*	33,084.8		33,029.9		13,247.0	

*Model 3*: year of birth + level of education + family-background characteristics.^4^

*Model 4*: year of birth + level of education + family fixed characteristics.

^1^ The bootstrap procedure was used to calculate the confidence intervals.

^2^ Omitted category in parentheses.

^3^ Only mothers included in the model.

^4^ In Model 3 number of siblings coded as 1–2 or 3– siblings.

*Source*: As for Table 1.

We also conducted corresponding fixed-effects regression analyses regarding having any children and the number of children beyond the first one (Table 5). The educational gradient in the fixed-effects subsamples is attenuated in both outcomes compared to the whole sample. For having any children (*n* = 4,491) the OR of those with a tertiary education is 0.86 (95 per cent CI 0.74, 1.00) compared to those with a basic education, and including the measured family-background variables (Model 3) or family fixed characteristics (Model 4) hardly changed the gradient. In the case of the fixed-effects subsample for the number of children beyond the first child (*n* = 11,569) the educational pattern is similar in direction to that in the whole sample: those with a tertiary education have as many children as those with a basic education (IRR 1.00 95 per cent CI 0.93, 1.07) and those with a secondary education correspondingly the fewest. As in the whole sample of women, here, too, adjustment for the measured family-background variables has an attenuating effect: the IRR of those with an upper-secondary education changes from 0.94 (95 per cent CI 0.89, 1.01) to 0.98 (95 per cent CI 0.92, 1.05). Adjustment for family fixed characteristics also attenuates the small educational differences in the number of children beyond the first one.

It is reasonable to expect that a large proportion of births to relatively young women are unplanned (Henshaw 1998; Vikat et al. 2002), and unplanned births may contribute to excess fertility among young women with a low level of education. In order to assess the effect of births at a young age (assuming they may be largely unplanned) on the educational gradient we analysed a subsample of women, excluding those who had their first child before turning 20 (14.6 per cent of mothers) (details for those available on request). The educational differences in fertility are not statistically significant in this subsample (*n* = 30,884): the year-of-birth-controlled IRR of those educated to the tertiary level is 0.98 (95 per cent CI 0.96, 1.01) compared to those with a basic education. However, the effect of adjusting for observed family-background characteristics in this subsample is similar to that among all women: the IRR of the former group rises to 1.01 (95 per cent CI 0.98, 1.04).

## Discussion

### Interpretation of the main results

We studied the association between women's own level of education and lifetime fertility in a cohort of Finnish females born in the years 1940–50, and whether observed or unobserved family-background characteristics contributed to this association. Our large register-based data set was drawn from the Finnish Census of Population in 1950, which provides for extensive follow-up and the identification of links to sisters. As expected, we found a negative association between a woman's educational level and her fertility: those with a basic education had a birth-year-adjusted fertility of 1.94 children, compared with 1.73 among those with a tertiary education. We also found a negative educational gradient for the likelihood of having any children, but there was a U-shaped association for fertility beyond the first child. Living with fewer siblings, in less crowded and rented housing, and in more urbanized areas in childhood predicted lower lifetime fertility, net of the effect of other family-background characteristics and individual educational attainment.

The observed family-background characteristics, mainly parents' socio-economic position, moderately accounted for the association between level of education and number of children. For example, the difference in lifetime fertility between women with a tertiary and a basic level of education decreased by 28 per cent following adjustment for the measured family-background characteristics. Analyses of childlessness and fertility beyond the first child showed that this was mainly because family background influenced the association between education and fertility beyond the first child. The results of the family fixed-effects models supported this interpretation.

These findings are consistent with those of other studies which, using varying fertility outcomes and analytical methods, report educational differences in fertility even after adjustment for family-background factors such as the number of siblings, parents' class, and the level of urbanization in childhood (e.g., Liefbroer and Corijn 1999; Lappegård and Rønsen 2005; Kravdal 2007; Kravdal and Rindfuss 2008, p. 865; Tavares 2010). The application of a simultaneous modelling technique that allows for correlated unobserved heterogeneity between education and childbearing has produced results broadly similar to those reported here and has also led to the conclusion that the outcomes may be partly jointly determined (Upchurch et al. 2002; Billari and Philipov 2004; Martín-García and Baizán 2006). However, studies based on quasi-experimental designs have produced less consistent evidence of a relationship between education and number of children (Kohler and Rodgers 2003; Skirbekk et al. 2006; Monstad et al. 2008; Kohler et al. 2010; Nisén et al. 2013).

Cohort fertility in Finland fell well below replacement level in the birth cohorts of females born in the late 1930s and 1940s, and the rising level of education for females may have contributed to this change (e.g., Andersson et al. 2009; Bhrolcháin and Beaujouan 2012). At the individual level the lower lifetime fertility of highly educated women may have been in part because spending a longer time studying in early adulthood required the postponement of family formation, and eventually led to some women remaining childless. On the other hand, it is possible that early childbearing inhibited others from studying for as long or as intensively as their childless peers.

Previous research has shown that in Finland at the end of the twentieth century, net of the effect of enrolment, education was positively associated with fertility rates among women aged 30 and over whereas among younger women those educated to the lowest level were at the highest risk of having a first child (Vikat 2004; Jalovaara and Miettinen 2013). In our data the gradient according to the highest degree attained at age 30–34 in age-specific fertility rate turned from negative to positive before age 30 and stayed positive at higher ages (the results are not shown but are available on request). Thus it seems that at higher ages highly educated Finnish women speed up (e.g., because of an income effect or awareness of decreasing fecundity) rather than hold up (e.g., because of opportunity costs) their childbearing.

A further analysis conducted on a subsample of women who remained childless until the age of 20 clearly showed smaller educational differences in lifetime fertility in general. It therefore seems that, regardless of the direction of the potential causal effect, the early adult years play a critical role in the determination of a negative gradient in women's lifetime fertility. This is in line with previous research showing that educational differences in women's cumulative fertility are at their largest in the Nordic countries in early adulthood: higher fertility rates among highly educated women at higher ages contribute to modest social differences in completed cohort fertility (Andersson et al. 2009). Because a large share of births at younger ages are likely to be unplanned (Henshaw 1998; Vikat et al. 2002), it may be that unplanned births have contributed to the negative gradient in lifetime fertility among Finnish women.

During the decades in which the women in the birth cohort we studied were having their children, Finnish society was moving towards the Nordic welfare-state model characterized by a high level of gender equality and generous state policies for families with children (Rønsen 2004; Rønsen and Skrede 2010). In consequence, the resources of the family of origin might have had relatively little effect on how the women ended up combining their family, educational, and occupational careers (Billari and Philipov 2004). For example, the moderate contribution of family background to the association between education and fertility beyond the first child may be attributable either to the timing of fertility (if daughters of well-off parents planned to attain both higher education and have children later this may have resulted in lower lifetime fertility for reasons clarified earlier) or to preferences for the eventual number of children. Finally, family background was a general determinant of lifetime fertility, as evidenced by the several associations found with socio-demographic family-background characteristics, some of which were also net of the effect of women's own achieved level of education.

### Study design considerations

The main strength of our study was that the analysis was based on an internationally unique data set that monitored events over a period long enough to allow the analysis of lifetime fertility, a large sample size without self-selection, and reliable information on childhood living conditions. Another unique advantage of monitoring events over such a long period is that the measured family-background variables were not retrospective or self-reported. There were very few missing values, reporting bias was likely to have been minimal, and we were able to adjust the education–fertility associations for a large number of well-measured socio-demographic confounders. Nevertheless, even the rich census information that we had from the year 1950 does not of course include all the family-background characteristics that may have influenced the fertility–education gradient.

In order to assess the contribution of unobserved family-background characteristics we applied family fixed-effects models that account for all unobserved family characteristics shared by sisters. However, even if unobserved shared family background is controlled for, it is still possible for unshared factors to confound the associations: for example, we were unable to control for unshared genetic characteristics and changes in family environment (e.g., Holmlund 2005; Kohler et al. 2010). Future studies of the causal relations between education and fertility could focus on such factors.

A caveat in fixed-effects analysis is the low statistical power resulting from the small proportion of the variance in the explanatory variable found within families (Allison 2009). In this study, 29.7 per cent of the total variance in the level of education among the women included in the Poisson fixed-effects model for the number of children (*n* = 15,746) was within families (between sisters). Thus, we consider the fixed-effects procedure was a feasible statistical tool here.

One could further question the external validity of the estimates owing to the selection of the sample in the fixed-effects analyses compared to conventional statistical models. An obvious consequence of the procedure is that women in the fixed-effects subsample have higher fertility levels and a lower educational level than women in the total sample. We excluded sister sets in which none had any children and, in the analysis of having any children, those sets in which all sisters had the same outcome (no/any children). Thus, the analysed subsamples consisted of sister sets in which fertility outcomes were somewhat more heterogeneous than among all sister sets. Similarity between sisters in an outcome variable, however, can be interpreted as a family influence on this variable as such (e.g., Kohler and Rodgers 2003).

In general, findings related to educational differences in fertility are sensitive to issues of measurement (Kravdal 2007). Owing to lack of data we were not able to distinguish between the associations of fertility rates with both educational enrolment and educational attainment (Hoem and Kreyenfeld 2006; Kravdal 2007). For reasons of clarity we used the level measured at age 30–34, which generally represents the final level of education a woman achieves during her lifetime (8.3 per cent of the women achieved a higher level by age 45–49). This choice may have contributed to an overestimation of a negative effect of education on fertility if many childless women acquired further qualifications at relatively high ages. With regard to the possible causal relationship between education and fertility, we made no strong assumptions on the direction of the effect. Given that our focus was on whether family background contributed to the association between education and fertility, we consider this measurement issue to have been a less serious weakness than it would be for other research questions.

## Conclusions

We analysed the influence of family-background characteristics on the association between education and lifetime fertility among Finnish women born in the years 1940–50. A higher level of personal education and several indicators of socio-demographic characteristics predicted lower fertility: women whose parents had a higher socio-economic position, a higher standard of living in childhood, fewer siblings, and an urban background had fewer children. Those with the lowest and highest levels of education, however, were relatively similar in fertility beyond the first child. Analyses controlling for observed and unobserved family characteristics indicated that family background moderately contributed to the trend among highly educated women to have fewer children, but not to their being less likely to have children in the first place. Thus, despite being a significant predictor of women's lifetime fertility overall, family background may explain only a small part of the education–fertility association. Further research is needed to analyse whether the association that remains net of the effect of the family-background characteristics captured in this study represents causal mechanisms running from fertility to education, or vice versa.

## References

[cit0001] Allison Paul D. (2009). *Fixed Effects Regression Models*.

[cit0002] Andersson Gunnar, Rønsen Marit, Knudsen Lisbeth B., Lappegård Trude, Neyer Gerda, Skrede Kari, Teschner Karin, Vikat Andres (2009). Cohort fertility patterns in the Nordic countries. *Demographic Research*.

[cit0003] Axinn William G., Clarkberg Marin E., Thornton Arland (1996). Family influences on family size preferences. *Demography*.

[cit0004] Becker Gary S. (1991). *A Treatise on the Family*.

[cit0005] Becker Gary S., Lewis H. Gregg (1973). On the interaction between the quantity and quality of children. *Journal of Political Economy*.

[cit0006] Bhrolcháin Máire Ni, Beaujouan Éva (2012). Fertility postponement is largely due to rising educational enrolment. *Population Studies*.

[cit0007] Billari Francesco C., Philipov Dimiter (2004). Education and the transition to motherhood: a comparative analysis of Western Europe. *European Demographic Research Paper* 2004/3.

[cit0008] Billari Francesco C., Goisis Alice, Liefbroer Aart C., Settersten Richard A., Aassve Arnstein, Hagestad Gunhild, Spéder Zsolt (2011). Social age deadlines for the childbearing of women and men. *Human Reproduction*.

[cit0009] Blossfeld Hans-Peter, Huinink Johannes (1991). Human capital investments or norms of role transition? How woman's schooling and career affect the process of family formation. *American Journal of Sociology*.

[cit0010] Carpenter James, Bithell John (2000). Bootstrap confidence intervals: when, which, what? A practical guide for medical statisticians. *Statistics in Medicine*.

[cit0011] Cohen Joel E., Kravdal Øystein, Keilman Nico (2011). Childbearing impeded education more than education impeded childbearing among Norwegian women. *Proceedings of the National Academy of Sciences*.

[cit0012] Dearden Kirk A., Hale Christiane B., Woolley Thomas (1995). The antecedents of teen fatherhood: a retrospective case-control study of Great Britain youth. *American Journal of Public Health*.

[cit0013] Easterlin Richard A. (1966). On the relation of economic factors to recent and projected fertility changes. *Demography*.

[cit0014] Fieder Martin, Huber Susanne (2007). The effects of sex and childlessness on the association between status and reproductive output in modern society. *Evolution and Human Behavior*.

[cit0015] Geronimus Arline T., Korenman Sanders (1992). The socioeconomic consequences of teen childbearing reconsidered. *Quarterly Journal of Economics*.

[cit0016] Gustafsson Siv (2001). Optimal age at motherhood. Theoretical and empirical considerations on postponement of maternity in Europe. *Journal of Population Economics*.

[cit0017] Gustafsson Siv, Kalwij Adriaan (2006). *Education and Postponement of Maternity. Economic Analyses for Industrialized Countries*.

[cit0018] Hagestad Gunhild O., Call Vaughn R. A. (2007). Pathways to childlessness: a life course perspective. *Journal of Family Issues*.

[cit0019] Hakim Cathrine (2000). *Work-Lifestyle Choices in the 21st Century. Preference Theory*.

[cit0020] Henshaw Stanley K. (1998). Unintended pregnancy in the United States. *Family Planning Perspectives*.

[cit0021] Hoem Jan M. (1986). The impact of education on modern family-union initiation. *European Journal of Population*.

[cit0022] Hoem Jan M., Kreyenfeld Michaela (2006). Anticipatory analysis and its alternatives in life-course research. Part 1: education and first childbearing. *Demographic Research*.

[cit0023] Hoem M. Jan, Neyer Gerda, Andersson Gunnar (2006a). Educational attainment and ultimate fertility among Swedish women born in 1955–59. *Demographic Research*.

[cit0024] Hoem Jan M., Neyer Gerda, Andersson Gunnar (2006b). The relationship between educational field, educational level, and childlessness among Swedish women born in 1955–59. *Demographic Research*.

[cit0025] Hoffman Saul D., Foster E. Michael, Furstenberg Frank F. (1993). Reevaluating the costs of teenage childbearing. *Demography*.

[cit0026] Hofferth Sandra L., Reid Lori, Mott Frank L. (2001). The effects of early childbearing on schooling over time. *Family Planning Perspectives*.

[cit0027] Holmlund Helena (2005). Estimating long-term consequences of teenage childbearing: an examination of the siblings approach. *The Journal of Human Resources*.

[cit0028] Jalovaara Marika (2012). Socio-economic resources and first-union formation in Finland, cohorts born 1969–81. *Population Studies*.

[cit0029] Jalovaara Marika, Miettinen Anneli (2013). Does his paycheck also matter? The socioeconomic resources of co-residential partners and entry into parenthood in Finland. *Demographic Research*.

[cit0030] Jäntti Markus, Saari Juho, Vartiainen Juhana (2006). Growth and equity in Finland.

[cit0031] Keizer Renske, Dykstra Pearl A., Jansen Miranda D. (2008). Pathways into childlessness: evidence of gendered life course dynamics. *Journal of Biosocial Science*.

[cit0032] Kneale Dylan, Joshi Heather (2008). Postponement and childlessness – evidence from two British cohorts. *Demographic Research*.

[cit0033] Kohler Hans-Peter, Behrman Jere R., Schnittker Jason (2010). Social science methods for twin data: integrating causality, endowments and heritability.

[cit0034] Kohler Hans-Peter, Rodgers Joseph L., Wachter Kenneth W., Bulatao Rodolfo A. (2003). Education, fertility, and heritability: explaining a paradox. *Offspring: Human Fertility Behavior in Biodemographic Perspective*.

[cit0035] Kravdal Øystein (2001). The high fertility of college educated women in Norway. An artefact of the separate modelling of each parity transition. *Demographic Research*.

[cit0036] Kravdal Øystein (2007). Effects of current education on second- and third birth rates among Norwegian women and men born in 1964: substantive interpretations and methodological issues. *Demographic Research*.

[cit0037] Kravdal Øystein, Rindfuss Ronald R. (2008). Changing relationships between education and fertility – a study of women and men born 1940–64. *American Sociological Review*.

[cit0038] Kreyenfeld Michaela (2002). Time-squeeze, partner effect or self-selection? An investigation into the positive effect of women's education on second birth risks in West Germany. *Demographic Research*.

[cit0039] Kulu Hill, Vikat Andres, Andersson Gunnar (2007). Settlement size and fertility in the Nordic countries. *Population Studies*.

[cit0040] Lahey Benjamin B., D'Onofrio Brian M. (2010). All in the family: comparing siblings to test causal hypotheses regarding environmental influence on behavior. *Current Directions in Psychological Science*.

[cit0041] Lappegård Trude, Rønsen Marit (2005). The multifaceted impact of education on entry into motherhood. *European Journal of Population*.

[cit0042] Lee Dohoon (2010). The early socioeconomic effects of teenage childbearing: a propensity score matching approach. *Demographic Research*.

[cit0043] Lestaeghe Ron (1983). A century of demographic and cultural change in Western Europe: an exploration of underlying dimensions. *Population and Development Review*.

[cit0044] Liefbroer Aart C., Corijn Martine (1999). Who, what, where, and when? Specifying the impact of educational attainment and labour force participation on family formation. *European Journal of Population*.

[cit0045] Lyngstad Torkild H., Jalovaara Marika (2010). Review of the antecedents of union dissolution. *Demographic Research*.

[cit0046] Martín-García Teresa, Baizán Pau (2006). The impact of the type of education and of educational enrolment on first births. *European Sociological Review*.

[cit0047] McElroy Susan W. (1996). Early childbearing, high school completion, and college enrollment: evidence from 1980 high school sophomores. *Economics of Education Review*.

[cit0048] Miller Warren B. (1992). Traits and developmental experiences as antecedents of childbearing motivation. *Demography*.

[cit0049] Miller Warren B. (1994). Childbearing motivations, desires, and intentions: a theoretical framework. *Genetic, Social and General Psychology Monologues*.

[cit0050] Moffitt Robert (2005). Remarks on the causal relationship in population research. *Demography*.

[cit0051] Moffitt Robert, Engelhardt Henriette, Kohler Hans-Peter, Fürnkranz-Prskawetz Alexia (2009). Issues in the estimation of causal effects in population research, with an application to the effects of teenage childbearing. *Causal Analysis in Population Studies. Concepts, Methods, Applications*.

[cit0052] Monstad Karin, Carol Propper, Salvanes Kjell G. (2008). Education and fertility: evidence from a natural experiment. *Scandinavian Journal of Economics*.

[cit0053] Morgan S. Philip, Rindfuss Ronald R. (1999). Reexamining the link of early childbearing to marriage and to subsequent fertility. *Demography*.

[cit0054] Murphy Michael, Knudsen Lisbeth B. (2002). The intergenerational transmission of fertility in contemporary Denmark: the effects of number of siblings (full and half), birth order, and whether male or female. *Population Studies*.

[cit0055] Nettle Daniel, Pollet Thomas V. (2008). Natural selection on male wealth in humans. *The American Naturalist*.

[cit0056] Nisén Jessica, Martikainen Pekka, Kaprio Jaakko, Silventoinen Karri (2013). Educational differences in completed fertility: a behavioral genetic study of Finnish male and female twins. *Demography*.

[cit0100] Oppenheimer Valerie. K (1997). Women's employment and the gain to marriage: the specialization and trading model. *Annual Review of Sociology*.

[cit0057] Parr Nicholas J. (2005). Family background, schooling and childlessness in Australia. 2005. *Journal of Biosocial Science*.

[cit0058] Pouta Anneli, Järvelin Marjo-Riitta, Hemminki Elina, Sovio Ulla, Hartikainen Anna-Liisa (2005). Mothers and daughters: intergenerational patterns of reproduction. *European Journal of Public Health*.

[cit0059] Ribar David C. (1999). The socioeconomic consequences of young women's childbearing: reconciling disparate evidence. *Journal of Population Economics*.

[cit0060] Rijken Arieke J., Liefbroer Aart C. (2009). Influences of the family of origin on the timing and quantum of fertility in the Netherlands. *Population Studies*.

[cit0061] Rindfuss Ronald R., Bumpass Larry L. (1976). How old is too old? Age and the sociology of fertility. *Family Planning Perspectives*.

[cit0062] Rindfuss Ronald R., Morgan S. Philip, Offutt Kate (1996). Education and the changing age pattern of American fertility: 1963–1989. *Demography*.

[cit0063] Rønsen Marit (2004). Fertility and public policies – evidence from Norway and Finland. *Demographic Research*.

[cit0064] Rønsen Marit, Skrede Kari (2010). Can public policies sustain fertility in the Nordic countries? Lessons from the past and questions for the future. *Demographic Research*.

[cit0065] Rønsen Marit, Sundström Marianne (2002). Family policy and after-birth employment among new mothers – a comparison of Finland, Norway and Sweden. *European Journal of Population*.

[cit0101] Scott J (2004). Family, gender, and educational attainment in Britain: A longitudinal study. *Journal of Comparative Family Studies*.

[cit0066] Skirbekk Vegard, Kohler Hans-Peter, Prskawetz Alexia, Gustafsson Siv, Kalwij Adriaan (2006). The marginal effect of school leaving age on demographic events. A contribution to the discussion on causality. *Education and Postponement of Maternity. Economic Analyses for Industrialized Countries*.

[cit0067] StataCorp (2009). *Stata Statistical Software: Release 11*.

[cit0068] Statistics Finland (1989). *Koulutusluokitus 31.12.1988. Käsikirjoja 1* [Finnish Standard Classification of Education 31.12.1988. Handbooks].

[cit0069] Statistics Finland (1997a). *Vuoden 1950 väestölaskennan otosaineiston käsikirja* [Handbook of the 1950 Census Sample].

[cit0070] Statistics Finland (1997b). *ISCED 1997. The Finnish Implementation Manual. Appendix to the Finnish Standard Classification of Education 1997. Handbooks*.

[cit0071] Tavares Lara (2010). Who Delays Childbearing? The Relationships between Fertility, Education and Personality Traits.

[cit0072] Thornton Arland (1980). The influence of first generation fertility and economic status on second generation fertility. *Population and Environment*.

[cit0073] Toulemon Laurent, Lapierre-Adamcyk Évelyne, Bledsoe Caroline, Lerner Susana, Guyer Jane I. (2000). Demographic patterns of motherhood and fatherhood in France. *Fertility and the Male Life-Cycle in the Era of Fertility Decline. International Studies in Demography*.

[cit0074] Upchurch Dawn M., Lillard Lee A., Panis Constantijn W. A. (2002). Nonmarital childbearing: influences of education, marriage and fertility. *Demography*.

[cit0075] van de Kaa Dirk J. (1996). Anchored narratives: the story and findings of half a century of research into the determinants of fertility. *Population Studies*.

[cit0076] Vikat Andres (2004). Women's labour force attachment and childbearing in Finland. *Demographic Research*.

[cit0077] Vikat Andres, Rimpelä Arja, Kosunen Elise, Rimpelä Matti (2002). Sociodemographic differences in the occurrence of teenage pregnancies in Finland in 1987–1998: a follow up study. *Journal of Epidemiology and Community Health*.

[cit0078] Weeden Jason, Abrams Michael J., Green Melanie C., Sabini John (2006). Do higher-status people really have fewer children? Education, income, and fertility in the contemporary U.S. *Human Nature*.

[cit0079] Woodward Lianne J., Fergusson David M., Horwood L. John (2006). Gender differences in the transition to early parenthood. *Development and Psychopathology*.

